# Visualising the invisible: a network approach to reveal the informal social side of student learning

**DOI:** 10.1007/s10459-012-9349-0

**Published:** 2012-02-01

**Authors:** J. Hommes, B. Rienties, W. de Grave, G. Bos, L. Schuwirth, A. Scherpbier

**Affiliations:** 1 Department of Educational Development and Research, Faculty of Health Medicine and Life Sciences, Maastricht University, Post Box 616, 6200 MD Maastricht, The Netherlands; 2 Centre for Educational and Academic Development, University of Surrey, Guildford, UK; 3 Department of Internal Medicine, Haematology, University Hospital Maastricht, Maastricht, The Netherlands; 4 Flinders Innovation and Clinical Education, Flinders Medical School, Flinders University, Adelaide, Australia

**Keywords:** Collaborative learning, Cooperative learning, Informal learning, Non-formal learning, Social networks, Student learning

## Abstract

World-wide, universities in health sciences have transformed their curriculum to include collaborative learning and facilitate the students’ learning process. Interaction has been acknowledged to be the synergistic element in this learning context. However, students spend the majority of their time outside their classroom and interaction does not stop outside the classroom. Therefore we studied how informal social interaction influences student learning. Moreover, to explore what really matters in the students learning process, a model was tested how the generally known important constructs—prior performance, motivation and social integration—relate to informal social interaction and student learning. 301 undergraduate medical students participated in this cross-sectional quantitative study. Informal social interaction was assessed using self-reported surveys following the network approach. Students’ individual motivation, social integration and prior performance were assessed by the Academic Motivation Scale, the College Adaption Questionnaire and students’ GPA respectively. A factual knowledge test represented student’ learning. All social networks were positively associated with student learning significantly: friendships (β = 0.11), providing information to other students (β = 0.16), receiving information from other students (β = 0.25). Structural equation modelling revealed a model in which social networks increased student learning (r = 0.43), followed by prior performance (r = 0.31). In contrast to prior literature, students’ academic motivation and social integration were not associated with students’ learning. Students’ informal social interaction is strongly associated with students’ learning. These findings underline the need to change our focus from the formal context (classroom) to the informal context to optimize student learning and deliver modern medics.

## Introduction

Currently, modern learning environments in health sciences build on three elements: ‘contextual’, ‘constructivist’ and ‘collaborative’ learning. Research in cognitive psychology has given considerable insights on how ‘contextual learning’—learning within a relevant context—and ‘constructivist learning’—learning is an active constructive process—both enhance knowledge acquisition and retention (Simons et al. 2000). Although considerable positive effects of collaborative learning on a variety (medical) competences were found e.g. (Johnson and Johnson 2009; Michaelsen et al. 2008), we still have no full understanding on the mechanisms how these effects are reached. Social interaction was found to be a key element in collaborative learning among students in the formal context (see (Baker 1999; Jeong and Chi 2007) for an overview). But, it is unlikely that this complex process of collaboration between students stops *outside* the “classroom” (Morone and Taylor 2004). In other words, social interaction *outside* small groups or classroom is likely to influence students’ learning processes in addition to the formal education (Hafferty 1998). Some studies have claimed that informal or non-formal learning is important, but how important it is, and in what way it can exert its influence on student learning is unfortunately still unknown (Barron 2006; Bransford et al. 2005; Marsick and Watkins 2001).

Good indications that informal social interaction has a large influence on learning, can be derived from studies in anthropology, mathematics and economics, using the “network approach” [see (Katz et al. 2004) for an overview]. Networks are present everywhere, defined as links between telephone wires or brain cells for example (Scott 2000). *Social* networks are networks between persons, with a broad range of the type of interpersonal relations, such as communication ties (who talks to whom), formal ties (who reports to who) or affective or kinship ties (who likes whom) (Hatala 2006; Katz et al. 2004; Scott 2000). The network approach is used in many context, which converge on the notion that a persons’ behaviour is mainly the result of the web of relationships around him/her, as these relationships provide opportunities and impose constrains on people’s behaviour (Katz et al. 2004; Wellman 1988). In other words, the network approach views individuals as interdependent, taking into account a person’s resources, information flow and relationships. Using this network approach, quite a few studies have confirmed that persons with many connections are also the persons that learn the most or perform better within organizations (see (Cross 2000) for an overview). In medicine, the network approach has been used amongst others, to study the diffusion and dissemination of innovations. Coleman et al. (1966) was the first to find that the diffusion of a new tetracycline-based medication among physicians was based on the physician’s social network; the stronger the ties of a particular physician, the more likely the physician was to be an early user of this new drug (Coleman et al. 1966). More recent studies underlined the finding that social networks are critical for the sustainability of health care innovation (Fattore et al. 2009; Jippes et al. 2010). All these studies emphasize the informal social side of learning within organizations and medical practice. Does this also apply to students? In other words, do students learn from informal social interaction, besides the formal interaction (e.g. interaction in tutorial groups) that students are involved in?

Surprisingly, researchers in the educational field have not tested this idea en masse. To the best of our knowledge, only three studies have investigated the influence of social networks in face-to-face educational programmes on student learning (Baldwin et al. 1997; Mayer and Puller 2008; Thomas 2000). The first study (Baldwin et al. 1997) found that individuals, who had many friends among the 304 first year students within an Masters of Business Administration course, had the highest grades. The same was found for the students who asked many other students explicitly for advice or help. These results imply that social networks among fellow students, substantially influenced student learning in this MBA programme. Thomas (2000) studied how social networks influenced students’ feelings of integration and student persistence based on Tinto’s model. He found that social networks are a pool of social and academic resources for students. The most recent study, (Mayer and Puller 2008), studied students’ “friends” or social networks on Facebook.com within their university campus. These researchers found that the average friends’ grade point average (GPA) was strongly associated with his/her own GPA.

Besides not distinguishing formal and informal interaction between students, what was not addressed by the previous studies on social networks and learning is the connection with the generally known variables affecting student learning: students’ social integration, academic motivation and prior performance. These variables might confound the association between social networks and learning. For example, family and friends of first-year students were found to give a feeling of “social integration” within the students’ academic surroundings, which was in turn positively associated with higher performance (Severiens and Wolff 2008; Tinto 1975; Wilcox et al. 2005). Friendships might thus diffuse knowledge or affect student behaviour following the network theory, which was found by (Thomas 2000). At the same time, friendships might provide social support resulting in a context that facilitates learning. Therefore, social networks might confound with feelings of social integration while researching student learning.

Students’ individual *motivation to learn* is another strong predictor of student learning (Ryan and Deci 2000; Vallerand and Pelletier 1993). Recently, students’ academic motivation was shown to be socially constructed; e.g. social relationships such as peers influence students’ motivation to learn (Järvelä et al. 2010; Wentzel 2005). Another study showed that students with merely intrinsic motivation built different social networks compared to students who were mainly extrinsically motivated (Rienties et al. 2009). As such, social networks seem to be intertwined between student learning and academic motivation.

A third confounding variable is *prior performance*, as this variable is an important predictor of students’ university performance in medical students (Lumb and Vail 2004). The question remains if social networks induce learning directly, as Baldwin et al. (1997) study suggested, or if students built different social networks based on students’ performance, which might be the case in Mayer and Puller (2008) study and explains why the GPA’s among friends were so homogeneous.

Thus, there are several possible confounding variables that need to be taken into account while studying students’ social interaction and learning. At the same time, we also need to take into the account the reciprocal relationships between social networks student learning and the confounding variables in the analyses.

To conclude these paragraphs, there are several indications that collaboration between (medical) students in the informal context could increase student learning. However, there are socially constructed variables, e.g. motivation and integration that could have direct and indirect effects on student learning and social networks and have not been taken into account in other studies. As social networks have previously been shown to provide insights into one’s social relationships and performance in other disciplines, this method seems to be very suitable to study informal learning. Hence, we researched the following two questions to increase our understanding on what really matters in the students’ learning process using social network analysis: 1) To what extent do social networks increase student learning independently from academic motivation, social integration and prior performance? 2) How does the effect of social networks on students’ learning relate to the previously mentioned possible confounders?

## Method

### Setting

The study took place at Maastricht University medical school in The Netherlands. This school offers a six-year undergraduate-entry medical course with a Problem Based Curriculum since 1974 (Graaff and Post 1985). Due to the great number of students wanting to study medicine, students are selected through a national lottery system, with higher chances for students with a high Grade point average (GPA). Only students with a GPA of 8.0 or higher (range 0–10) can enter the medical school without having to take part in the lottery system (about 20% of the medical students). The first three years compromise the preclinical curriculum and is organised in modules of six to ten weeks. The basic element in these first three years is the tutorial groups. These groups consist of 8–11 students, resulting in a varying group composition after every module. Lectures, skill trainings and anatomy sessions complement the tutorial groups.

### Participants

The first year students (n = 301) were asked to participate into this study, after they had completed 10 months of their first year in our medical school.

### Tests and measurements

#### Social networks

A specific methodology has been developed for social network studies. This social networks methodology defines social networks as a set of actors or individuals (“nodes”) and the interrelationships (“ties”). Of course, networks can stretch almost indefinitely, therefore social network analysis requires the researchers to specify boundaries (Knoke and Yang 2008). For our study, we have focussed on the informal networks of first year medical students. In social network methodology, this is denoted as a closed egocentric network.

Three network types were assessed (see Table 1) relating to friendships, giving and receiving information, while providing students with a list of names from students within the complete year group, following Marsden (1990). To increase validity, explicit timing was included (e.g. “during this module, I gave information to the following students”). A pilot tested the survey.

**Table 1:** The three (translated) questions to collect the relational data. Students were provided with a list of the complete year group. Names could be added if the students were not found on this list

Please indicate which of your fellow students are good friends of yours. For example, people with whom you go for a coffee or to the theatre with
Please indicate which of your fellow students have been important sources of school-related information, outside the tutorial groups, yet during the current module. Getting information from another could have occurred in various ways, for example someone gave you a summary or shared the learning goals of his/her tutorial group
Please indicate to which of your follow students you have given school-related information outside the tutorial groups, yet during the current module. Providing information can occur, for example, if you have answered someone else’s questions

*Friendship* networks explore passive information diffusion, while communication networks have a more instrumental nature (e.g. asking explicitly for help on a certain topic) (Ibarra and Andrews 1993; Katz et al. 2004). Two *communication* networks were assessed: “giving information” and “getting information”. Receiving information was assessed since this directly represents information acquisition, while providing other students with information, was regarded as an active process of elaboration, which has been shown to induce learning (Webb et al. 1995).

Tie strength can cause differences in information flow (Grannovetter 1973). For example, “being friends” as a binary representations (yes/no), might differ among students due to differences in participants’ definition (Scott 2000). Therefore, just showing the ties between persons, while not taking into account the value of these ties, might result in a loss of valuable information and could even be misleading (Freeman 1991). Therefore, tie strength was assessed. In the communication networks, we assessed students’ *value of the information that was given or received*, measured on a Likert scale. The range to describe the value of the information was: ‘not really valuable’ (1) to ‘very valuable’ (5). In the friendship network we assessed the same dimension. However, due to the passive nature of the information diffusion, the value dimension was termed “intensity of the friendship” with a range: ‘not really intense’ (1) to ‘very intense’ (5).

#### Academic motivation

The students’ academic motivation was measured by the Academic Motivation Scale (AMS), developed by (Vallerand et al. 1992). This scale was developed for university students and consists of 28 items. The AMS is divided into seven subscales, of which three belong to intrinsic motivation scale, three to extrinsic motivation scale and one for a-motivation. These scales constitute a motivational continuum reflecting the degree of self-determined behaviour. The Cronbach alpha for the seven subscales ranged from 0.73 to 0.85, which is in line with previous studies (Rienties et al. 2009; Vallerand et al. 1992). A relative autonomy index (RAI) (Grolnick and Ryan 1987) was computed based on the seven scales. A high score reflects high autonomous academic motivation and has been used by several studies (Black and Deci 2000).

#### Social integration

We used the College Adaptation Questionnaire (CAQ), originally constructed in Dutch by (Crombag 1968). It is a self-reporting instrument and consists of 18 statements, scored on a 7-Likert scale (range: (1) ‘I do not agree at all’ to (7) ‘I fully agree’). Ten of the items reflect poor adjustment (e.g. “I find it hard to get used to life here”) and eight items reflect good adjustment (e.g. “I am glad that I came to study here”). The score for the CAQ is the sum score the items representing good adjustment, minus the sum score on the items indicating poor adjustment. Previous studies have reported the CAQ to be highly consistent, α = 0.83 (van Rooijen 1986). In this study the Cronbach’s alpha was 0.89.

#### Prior performance

In our study we have used the category indicator for the grade point average (GPA) achieved at the end of secondary school as a measure of prior performance.

#### Student learning

While students were explicitly asked with whom one shared information with, during one module, student’ learning was quantified by performance on the module test (1–10). This factual knowledge orientated test was a written exam, with multiple-choice questions. There were 15 themes, encompassing “unconsciousness”; including basic medical knowledge on physiology, pharmacology anatomy, clinical examination, radiology and pathology. The test items (n = 100) had a high reliability (Cronbach α = 0.79).

### Procedure

We approached the students during an obligatory curricular activity: the tutorial groups. If students were absent in their group, they were contacted through email and asked to fill in the questionnaire online. We used this approach, because missing data have considerable negative effects on social network analysis (Huisman 2007), since interpretations of social network relations rely heavily on the assumption that the presence or absence of ties are identified (Gile and Handcock 2006).

Two sessions, one week apart, were planned during the tutorial groups. During the first session, the academic motivation and academic integration scales were administered. In the penultimate week of the module, the social networks questionnaire was assessed. Both survey sessions lasted 15 min. First, written information on the aims of the research was given, followed by a description of the survey. Students were assured that the collected data were strictly confidential and only used for research purposes. Second, students were asked for written informed consent to participate.

Ethical approval was obtained from the educational management board of the Faculty as educational research is exempted from the Medical Ethics Committee and a national ethics committee for research in the field of medical educational was still under construction when this study commenced.

## Statistical analyses

Centrality is the construct we used to quantify the relatedness to other students in the social networks (Knoke and Yang 2008; Scott 2000). An actor’s centrality reflects one’s connectivity to other actors in the network and was calculated using UCINET (Borgatti et al. 2002), a social network software programme. From the various types of centrality that have been described in the literature, we used Freeman’s degree centrality for valued data (Freeman 1991). This type of centrality indicates that students with many & strong relations are the prominent actors in the network (Freeman 1978). To eliminate the effect of the network size, we used the normalised score, which divides the centrality score by the maximum number of possible connections with the other actors.

We visualised the social networks of students using Pajek (Bategelj and Mrvar 2010), a programme designed to graph large social networks. Using the ‘Kamada-Kawai’ algorithm, the nodes were projected in such manner that they hardly showed overlap, facilitating interpretability.

### Part 1: Social networks and student learning

Once centrality was calculated for all first year students in the “friendship”, “giving information” and “getting information” networks, regression analyses using STATA (StataCorp 2010) were used to detect if centrality within the social network increased students’ performance. The centrality measures in all three measures were used as the independent variables and students’ performance as the dependent variable. All analyses were controlled for social integration, academic motivation, prior performance, gender and age.

### Part 2: Understanding the association between social networks, motivation, integration, prior performance on student’ learning

**Fig. 1:**
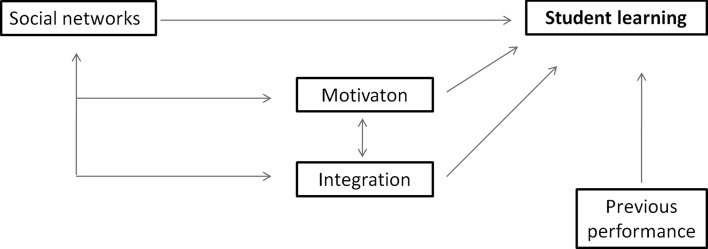
Social networks have been shown to increase student learning. In addition, academic motivation, social integration and prior performance have been shown increase student learning as well. In addition, motivation and social integration have shown to be socially constructed, which might cause indirect effects of social networks on student learning

To identify the relations between students’ performance, social integration, academic motivation, prior performance and social networks, as is depicted in Fig. 1, the structural equation modelling programme Amos (Amos Development Corporation 2008) was used.

Structural equation modelling combines multiple regression and path analysis to enable testing of the causal relations in a hypothetical model (MacKinnon 2008). Amos produces several goodness-of-fit criteria indicating how well the tested model “fits” the data, while taking into account the covariance and variance structures. The coherence of the following commonly used measures, indicated the model-fit (Humphris 2002; Violato and Hecker 2007):

- Chi square goodness of fit value provides a level of significance (*p*). In order not to reject the model, the *p* value should be higher than 0.05.
- Chi square (minimum discrepancy) divided by the degrees of freedom (CMIN/DF) should be less than 3 and preferably close to 1 for correct models.
- Comparative fit index (CFI) compares the fit of the model under test with a model in which none of the variables are related. A CFI value of ≥0.90 indicates that the model fits the data well.
- The root mean square error of approximation (RMSEA) includes the degrees of freedom, which might compensate for the effect of model complexity (Steiger 1990). This value is required to be smaller than 0.05.

## Results

From the 301 first year students (63.4% female), 276 students (91.7%) participated in the first session, while 260 students (85.7%) participated in the second session. We dealt with the missing relational data following (Gile and Handcock 2006), treating the missing ties on the precise estimates of mutuality and other (full) network characteristics to fit from the observed data.

No data were lost. The demographics are listed in Table 2.

**Table 2:** Demographics of the participants

	Mean	SD
Age (years)	20.0	1.21
Prior performance (1–5)	3.07	1.19
Factual knowledge test score (0–10)	7.10	0.99
Number of friends	9.42	9.39
Number of students giving information to	6.67	5.38
Number of students getting information from	3.73	3.00

### Part 1: Social networks and student learning

**Fig. 2:**
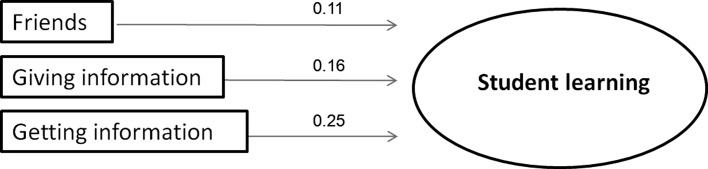
Centrality in the three social networks (friends, giving information and getting information from other students), increase student learning

All three social networks increased students’ performance on the module test, while statistically controlling for academic motivation, social integration, age, gender and prior performance (see Fig. 2): Centrality within the friends-network (β = 0.11, S.E. = 0.04 *p* = 0.012), centrality within the social network of providing information to fellow students (β = 0.16, S.E. = 0.05, *p* = 0.003) and centrality within the network of students receiving information (β = 0.25, S.E. = 0.06, *p* < 0.001). These findings cannot be explained by compensation behaviour of students using informal social interaction if they did not learn enough in their (formal) tutorial groups, as there was no (significant) association between the average students’ perception of their tutorial group effectiveness and all three students’ social network centralities.

**Fig. 3:**
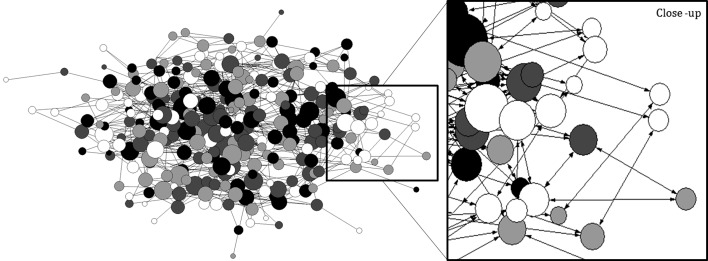
Visualization of how receiving information increase student learning. This figure visualizes how students receive information from fellow students (‘Get’ network). Students that learned most are positioned more in the centre of the network and have more connections to others compared to students that learned less.The nodes represent the students and the arrows show the information flow among the students. The larger the node, the more information the student has gathered from other students and the more valuable the information. The colour of the node indicates the performance on the factual knowledge test. ‘Black’ nodes indicate the students with the highest quartile scores on the knowledge test (smart students), dark and light ‘gray’ represent the intermediate two quartiles and ‘white’ nodes represent the lowest scoring students (least smart students)

The effects of social networks on student learning are also visually illustrated in Fig. 3.

### Part 2: Understanding the association between social networks, motivation, integration, prior performance on student’ learning

**Fig. 4:**
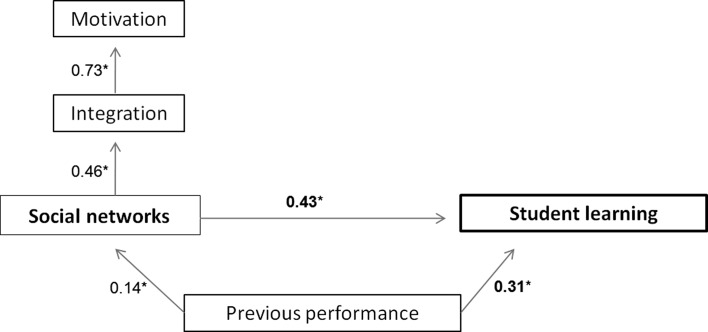
The modified model on the associations between social network and its confounders on students’ performance, *p < 0.001

The model (Fig. 1) consisted of paths from the independent variables social networks, academic motivation, social integration and prior performance; to the dependent variable, students’ performance. The independent variable social networks represented a latent variable, composed of all three networks (friendships, giving and getting information). The model fit indicators showed that the model could use improvement (χ^2^ = 31.38, *p* = 0.003, CMIN/df = 2.14, CFI = 0.95, RMSEA = 0.07). Modifications were made using a model trimming method (Ambramson et al. 2005; Ullman 2006). First, all theoretical paths were addressed to over-fit the model. Then, one parameter at a time was changed based on the modification indexes. Modifications of the proposed model were made based on theoretical possibilities, starting with removing the associations of academic motivation and social integration on student learning. Next, academic motivation was not associated with social networks, instead it was only associated with social integration (r = 0.73, S.E. = 0.11, *p* < 0.01). Finally, prior performance was found to influence not only students’ performance, but also students’ centrality within the social networks (r = 0.14, S.E. = 0.04, *p* < 0.001). The modified model, as can be seen in Fig. 4, fitted the data quite well (χ^2^ = 18.91, *p* = 0.22, CMIN/dif = 1.26, CFI = 0.99, RMSEA = 0.03).

Although social networks were not associated with academic motivation, these networks did associate positively with students’ social integration (r = 0.46, S.E. = 0.13, *p* < 0.001). Moreover, social networks showed to have greater effects on student learning than prior performance (r = 0.43, S.E. = 0.08, *p* < 0.001 and r = 0.31, S.E. = 0.04, *p* < 0.001, respectively).

## Discussion

World-wide education in health sciences involves collaborative learning techniques. While positive effects have been reported of this learning context, we researched the informal side of group interaction and student learning. In more concrete terms, this study assessed whether the informal social interaction or ‘social networks’ among undergraduate medical students, increased learning. A related research question was how other important influences on student learning; such as academic motivation, social integration and prior performance were related.

All three social networks increased student’ learning significantly, even after controlling for academic motivation, social integration, age, gender and prior performance. This finding is not only in line with the notion that learning in inherently social (Levine and Resnick 1993), but more specifically with the literature stating that social interaction is a key element in the (collaborative) learning processes e.g. (Larson 2009). Moreover, to the best of our knowledge, we are the first to quantify the impact of informal social interaction on learning. These results might make more sense while incorporating the wide variety of phenomena in which students might interact informally. Students might ask others explicitly for advice or help to complete learning goals or assignments. Yet, it might also consist of sharing (elaborations of) learning goals with students from other tutorial groups in order to ensure that their own group is on the right track. At the same time, passive knowledge diffusion occurs when reflecting one’ activities, thoughts and university related experiences with friends.

A new model was identified in our search how the various socially important variables (academic motivation & social integration) were associated with student learning. In this model, social networks were the strongest predictors for student learning followed by prior performance. In turn, prior performance was predictive of the strength of the social networks, indicating that the students with high prior performance were more central in the three social networks studied. In contrast to other studies (Severiens and Wolff 2008; Vallerand and Pelletier 1993), academic motivation and social integration were not predictive for student learning. Of course this could be explained by the fact that we are the first to include all variables in one model, instead of focusing on the relation between integration *or* motivation *and* student learning. This is supported by the finding that we did find a significant, but small association between academic motivation or social integration on student learning while omitting social networks in these analyses (β = 0.04, S.E. 0.02, *p* = 0.033 and β = 0.09, S.E. 0.04, *p* = 0.023, respectively). Another reason could be a difference in setting and participants; for example, the studies on motivation and performance were mainly based on undergraduate students within the United States and not among students in Medical school in a European setting.

Three studies have suggested associations between learning and social networks within (formal) educational contexts (Baldwin et al. 1997; Mayer and Puller 2008; Thomas 2000). However, these studies did not make direct associations nor took into account other socially constructed variables, which are known to directly and indirectly influence student learning. As such, this study has added new insights into what really matters in student learning amongst medical students, mainly emphasizing the importance of social networks and informal learning. These results are important since policy makers and curriculum designers might facilitate the development of these networks and as such might be able to influence student learning. One measure that would be supported by our findings would be increasing the frequency of collaboration with the same students to strengthen ties between students. Especially in large scale schools, with numbers of students up to n = 600 in one class (Moust et al. 2005), it is clear that students spend limited time collaborating, because they change groups frequently. At a first glance, meeting so many students during the year might seem as an advantage since students can construct very large networks. However, a network with mainly weak ties has been shown not to increase performance (Cross and Thomas 2008). Increasing the frequency of students meeting one another might help increasing the strength of students networks due to the fact that spending time together already causes feelings of relatedness (Baumeister and Leary 1995). And just these feelings of relatedness are needed to help one another (Bell et al. 2001; Stewart-Williams 2007). The implications for a collaborative learning context is that these feelings of interdependence among the students are essential in order to engage them in intensive collaborative learning (Johnson et al. 2007).

Besides the new insights this study has provided, it also has limitations. First, the diffusion or exchange of information amongst the first year students is measured as a subjective factor. Inherently to this self-reported measurement, three types of biases could have occurred which can reduce the reliability of the construct. First, students might have filled in the surveys according to what they thought the researcher desired them to answer. Secondly, errors of commission could have occurred by students introducing non-existent ties or over-estimations of their relationship(s). Finally, students could have forgotten to include important relations, causing an error of omission. Nonetheless, it can be argued that a self-reported measure needs to be used in this study as it captures students’ perception of their social network. In order to study how (learning) behaviour is influenced by social networks, it is essential to measure the perception of the (informal) social network relations, as behaviour only changes as a reaction of the perceived environment.

Instead of using valued data in the analysis, some network researchers prefer to convert the data into binary values. Furthermore, some researchers prefer other centrality measures than degree centrality such as betweenness centrality. In contrast to degree centrality, this type of centrality does not focus on the number of connections to others; it focuses on the actor’s position within the network in the sense that it connects subgroups of students. In other words, if students withhold a brokers’ position, these students have higher “betweenness centrality” measures (Scott 2000). We tested if binary graphs or betweenness centrality would change our results and found that both the regression analyses as the model were similar to the described results. Next, student learning is represented as achievement by one knowledge test. This representation is, of course, too simplistic (Adams 2006) although most universities do heavily rely on these performance measures (Kuh et al. 2006). Since the test was acknowledged to be valid and reliable, we believe that this test was an appropriate representation of students’ learning. Finally, since this research used an egocentric network (assessing only social networks among first-year students), we might have only grasped the tip of the iceberg, since most students (68.3%) declared that they also shared information (on medical subjects) with others (e.g. family, mentor, medical students not in their first year).

Despite these empirical limitations, we feel that this study, contributes to the understanding of informal social interaction within learning. It indicates the importance of the informal side of social learning. As such, we may need to expand our focus to include the informal aspects of learning in the design and evaluation our education as was also suggested by Hafferty (1998). Future research will need to focus on how networks are constructed (why are students connected? How do students develop networks over time?) and what type of information is exchanged. At the same time, students in a PBL system are used to collaborate with fellow-students. In a more traditional curriculum the association between informal social interaction and learning might be different. Insights into the influences of learning contexts might be provided when similar studies are done in another learning context. Moreover, using mixed methods or qualitative designs, studies might provide insights how informal social interaction facilitate student learning. With all these insights, it might be feasible to understand how educational institutes can influence students’ social networks to increase learning. Next, studies may explore whether a change in educational design actually changes students’ social networks and in the end increases student learning. Furthermore, social networks might also be used as an action research tool to increase understanding on student learning. In organizations, social networks analysis provides insights which persons hold valuable information or key positions. In education, social networks, might provide insights in which students could use some extra help to improve their learning process. Finally, in medical practice social network analysis might give valuable insights on how students or graduates learn in the workplace.

## Conclusion

Social networks were positively associated with student learning, with greater strength than students’ prior performance, academic motivation and social integration.
